# Supplementary material for: MetaRibo-Seq measures translation in microbiomes
Source: Nat Commun. 2020 Jun 29;11:3268. doi: 10.1038/s41467-020-17081-z (PMC7324362; doi:10.1038/s41467-020-17081-z)
Supplement: Supplementary file 10 — Supplementary Data 7 [file 41467_2020_17081_MOESM10_ESM.zip › File2/Confidence_High_Taxonomy/231257_out.krona.html]

Javascript must be enabled to view this page.

members
magnitude
magnitudeUnassigned
count
unassigned
taxon
rank

231257\_out

352

superkingdom
352
2

976
352
phylum

200643
352
class

352
171549
order

family
171552
1

577309
1
genus


SRS048164\_contig\_number\_21491
454154
1
species

1853231
1
family

1
283168
genus


SRS050925\_contig\_number\_12704
28118
1
species

family
815
350


SRS050299\_contig\_number\_contig-100\_16013.46487SRS056259\_contig\_number\_26666SRS063985\_contig\_number\_31613SRS148319\_contig\_number\_contig-100\_9497.75558
350
4
816
genus


SRS019381\_contig\_number\_3044
1
387090
species

species

SRS063190\_contig\_number\_14157
1
817

species

SRS011084\_contig\_number\_35629SRS011134\_contig\_number\_6703SRS011239\_contig\_number\_5722SRS011405\_contig\_number\_14446SRS012849\_contig\_number\_12689SRS012902\_contig\_number\_379SRS012969\_contig\_number\_13461SRS013158\_contig\_number\_4568SRS013382\_contig\_number\_1389SRS013521\_contig\_number\_5484SRS013638\_contig\_number\_2795SRS013687\_contig\_number\_34573SRS013800\_contig\_number\_25761SRS014235\_contig\_number\_3518SRS014287\_contig\_number\_828SRS014313\_contig\_number\_597SRS014412\_contig\_number\_4031SRS014459\_contig\_number\_3036SRS014534\_contig\_number\_1467SRS014683\_contig\_number\_3054SRS014736\_contig\_number\_4165SRS014855\_contig\_number\_3513SRS014948\_contig\_number\_3268SRS014979\_contig\_number\_34536SRS015190\_contig\_number\_15788SRS015217\_contig\_number\_298SRS015264\_contig\_number\_9855SRS015369\_contig\_number\_5644SRS015431\_contig\_number\_19139SRS015486\_contig\_number\_203SRS015486\_contig\_number\_273SRS015663\_contig\_number\_5449SRS015694\_contig\_number\_12854SRS015782\_contig\_number\_4074SRS015854\_contig\_number\_11473SRS015960\_contig\_number\_1361SRS016056\_contig\_number\_1613SRS016095\_contig\_number\_2541SRS016132\_contig\_number\_2641SRS016203\_contig\_number\_544SRS016335\_contig\_number\_9773SRS016495\_contig\_number\_contig-100\_38.121836SRS016753\_contig\_number\_4389SRS016840\_contig\_number\_2235SRS016897\_contig\_number\_89SRS016954\_contig\_number\_26471SRS016989\_contig\_number\_2306SRS017191\_contig\_number\_2142SRS017247\_contig\_number\_6256SRS017307\_contig\_number\_1697SRS017433\_contig\_number\_12SRS017521\_contig\_number\_3314SRS017701\_contig\_number\_10686SRS017745\_contig\_number\_2123SRS017821\_contig\_number\_29887SRS017859\_contig\_number\_62SRS018351\_contig\_number\_2644SRS018427\_contig\_number\_30989SRS018623\_contig\_number\_1469SRS018817\_contig\_number\_1385SRS018836\_contig\_number\_26361SRS018888\_contig\_number\_6912SRS018936\_contig\_number\_120SRS019030\_contig\_number\_4746SRS019068\_contig\_number\_48912SRS019161\_contig\_number\_12278SRS019178\_contig\_number\_42SRS019267\_contig\_number\_1589SRS019397\_contig\_number\_2378SRS019445\_contig\_number\_2969SRS019496\_contig\_number\_2354SRS019601\_contig\_number\_1172SRS019693\_contig\_number\_1120SRS019808\_contig\_number\_12449SRS019910\_contig\_number\_17067SRS019968\_contig\_number\_17473SRS020233\_contig\_number\_52523SRS020328\_contig\_number\_377SRS020394\_contig\_number\_14324SRS020508\_contig\_number\_1097SRS020622\_contig\_number\_465SRS020869\_contig\_number\_35787SRS021153\_contig\_number\_1362SRS021219\_contig\_number\_1626SRS021484\_contig\_number\_5005SRS021948\_contig\_number\_451SRS022137\_contig\_number\_11824SRS022524\_contig\_number\_16291SRS023526\_contig\_number\_276SRS023829\_contig\_number\_49SRS023914\_contig\_number\_11975SRS023971\_contig\_number\_4742SRS024009\_contig\_number\_8574SRS024075\_contig\_number\_9722SRS024331\_contig\_number\_23449SRS024492\_contig\_number\_339SRS024625\_contig\_number\_307SRS042284\_contig\_number\_3215SRS042966\_contig\_number\_18074SRS043411\_contig\_number\_contig-100\_278.103477SRS043667\_contig\_number\_1646SRS043701\_contig\_number\_10581SRS044535\_contig\_number\_18472SRS045004\_contig\_number\_361SRS045244\_contig\_number\_259SRS045645\_contig\_number\_312SRS045739\_contig\_number\_1401SRS046369\_contig\_number\_4725SRS046810\_contig\_number\_contig-100\_25.26SRS047014\_contig\_number\_36080SRS047044\_contig\_number\_14547SRS047433\_contig\_number\_18840SRS048870\_contig\_number\_7608SRS049402\_contig\_number\_4347SRS049446\_contig\_number\_6327SRS049712\_contig\_number\_24602SRS049773\_contig\_number\_453SRS049896\_contig\_number\_7998SRS049900\_contig\_number\_12223SRS049959\_contig\_number\_16943SRS049995\_contig\_number\_24488SRS050026\_contig\_number\_315SRS050422\_contig\_number\_10536SRS050520\_contig\_number\_contig-100\_61.23502SRS050752\_contig\_number\_3767SRS050801\_contig\_number\_1598SRS050941\_contig\_number\_3089SRS050974\_contig\_number\_1817SRS050998\_contig\_number\_12974SRS051031\_contig\_number\_32867SRS051610\_contig\_number\_10940SRS051882\_contig\_number\_4096SRS052027\_contig\_number\_4732SRS052576\_contig\_number\_contig-100\_60.15569SRS052697\_contig\_number\_867SRS053087\_contig\_number\_2125SRS053214\_contig\_number\_2620SRS053335\_contig\_number\_2214SRS053356\_contig\_number\_12408SRS053398\_contig\_number\_19446SRS053649\_contig\_number\_14912SRS054059\_contig\_number\_5122SRS054352\_contig\_number\_11158SRS054905\_contig\_number\_1163SRS055017\_contig\_number\_contig-100\_167.197610SRS055966\_contig\_number\_173SRS055982\_contig\_number\_14315SRS056273\_contig\_number\_4466SRS056537\_contig\_number\_17458SRS057478\_contig\_number\_608SRS057480\_contig\_number\_5186SRS058145\_contig\_number\_contig-100\_9637.9638SRS058723\_contig\_number\_2243SRS062427\_contig\_number\_8274SRS062654\_contig\_number\_12887SRS062701\_contig\_number\_6181SRS063040\_contig\_number\_57SRS063127\_contig\_number\_1646SRS063370\_contig\_number\_851SRS063489\_contig\_number\_806SRS063518\_contig\_number\_17905SRS063813\_contig\_number\_4810SRS063928\_contig\_number\_497SRS064557\_contig\_number\_16161SRS064645\_contig\_number\_175SRS064973\_contig\_number\_358SRS065176\_contig\_number\_78SRS065397\_contig\_number\_1392SRS065504\_contig\_number\_4430SRS074586\_contig\_number\_contig-100\_766.55740SRS074964\_contig\_number\_601SRS075078\_contig\_number\_21589SRS075398\_contig\_number\_101SRS075716\_contig\_number\_contig-100\_59.43532SRS075773\_contig\_number\_8296SRS075821\_contig\_number\_2SRS075878\_contig\_number\_12129SRS075984\_contig\_number\_633SRS076756\_contig\_number\_9824SRS076876\_contig\_number\_230SRS076976\_contig\_number\_653SRS077086\_contig\_number\_5870SRS077194\_contig\_number\_20835SRS077231\_contig\_number\_25659SRS077294\_contig\_number\_8916SRS077454\_contig\_number\_contig-100\_457.116435SRS077502\_contig\_number\_1420SRS077552\_contig\_number\_13614SRS077589\_contig\_number\_563SRS077641\_contig\_number\_23411SRS077849\_contig\_number\_1935SRS077991\_contig\_number\_186SRS078176\_contig\_number\_10166SRS078242\_contig\_number\_11503SRS078665\_contig\_number\_647SRS097889\_contig\_number\_10020SRS097920\_contig\_number\_6354SRS097958\_contig\_number\_9990SRS098061\_contig\_number\_9030SRS098073\_contig\_number\_3857SRS098571\_contig\_number\_27844SRS098644\_contig\_number\_4293SRS098827\_contig\_number\_8691SRS098881\_contig\_number\_3836SRS100021\_contig\_number\_9897SRS100907\_contig\_number\_contig-100\_55.68591SRS101376\_contig\_number\_5689SRS101433\_contig\_number\_14700SRS101433\_contig\_number\_contig-100\_22182.62845SRS103987\_contig\_number\_25686SRS104036\_contig\_number\_contig-100\_650.40435SRS104084\_contig\_number\_2211SRS1041031\_contig\_number\_13638SRS1041037\_contig\_number\_4SRS1041090\_contig\_number\_1796SRS1041091\_contig\_number\_20319SRS1041092\_contig\_number\_1256SRS1041095\_contig\_number\_2410SRS1041116\_contig\_number\_7889SRS1041118\_contig\_number\_15730SRS1041129\_contig\_number\_14278SRS1041130\_contig\_number\_3039SRS1041133\_contig\_number\_27342SRS1041134\_contig\_number\_1197SRS1041138\_contig\_number\_13625SRS1041139\_contig\_number\_37SRS1041140\_contig\_number\_10459SRS1041141\_contig\_number\_10241SRS1041142\_contig\_number\_50SRS1041144\_contig\_number\_6655SRS1041147\_contig\_number\_1097SRS1041157\_contig\_number\_7167SRS1041164\_contig\_number\_1540SRS104327\_contig\_number\_5159SRS104400\_contig\_number\_23615SRS104485\_contig\_number\_219SRS104693\_contig\_number\_329SRS104912\_contig\_number\_5164SRS104975\_contig\_number\_15SRS105082\_contig\_number\_163SRS1054716\_contig\_number\_15029SRS1054928\_contig\_number\_79SRS1055022\_contig\_number\_1742SRS1055038\_contig\_number\_92SRS1055049\_contig\_number\_10718SRS1055056\_contig\_number\_12535SRS1055069\_contig\_number\_2001SRS1055076\_contig\_number\_128SRS1055083\_contig\_number\_13075SRS1055095\_contig\_number\_432SRS142503\_contig\_number\_17691SRS142542\_contig\_number\_1719SRS142599\_contig\_number\_23436SRS142781\_contig\_number\_81SRS142890\_contig\_number\_253SRS142923\_contig\_number\_13318SRS142980\_contig\_number\_210SRS143070\_contig\_number\_2460SRS143085\_contig\_number\_31SRS143148\_contig\_number\_6834SRS143181\_contig\_number\_14407SRS143372\_contig\_number\_1724SRS143417\_contig\_number\_3559SRS143722\_contig\_number\_1481SRS143780\_contig\_number\_17467SRS143876\_contig\_number\_11851SRS143895\_contig\_number\_31915SRS143991\_contig\_number\_14932SRS144183\_contig\_number\_29923SRS144297\_contig\_number\_2069SRS144362\_contig\_number\_9525SRS144506\_contig\_number\_2480SRS144537\_contig\_number\_30166SRS144603\_contig\_number\_3370SRS144714\_contig\_number\_16732SRS144753\_contig\_number\_contig-100\_1.92572SRS145308\_contig\_number\_2355SRS145497\_contig\_number\_11375SRS146764\_contig\_number\_2889SRS146813\_contig\_number\_3962SRS147022\_contig\_number\_190SRS147039\_contig\_number\_2848SRS147271\_contig\_number\_6128SRS147346\_contig\_number\_1463SRS147425\_contig\_number\_879SRS147614\_contig\_number\_3299SRS147766\_contig\_number\_2508SRS147977\_contig\_number\_1171SRS148091\_contig\_number\_14015SRS148196\_contig\_number\_29767SRS148253\_contig\_number\_10SRS148424\_contig\_number\_2911SRS148511\_contig\_number\_17270SRS148721\_contig\_number\_17365SRS148817\_contig\_number\_1408SRS148970\_contig\_number\_28SRS149075\_contig\_number\_984SRS149181\_contig\_number\_1909SRS149244\_contig\_number\_3366SRS149325\_contig\_number\_13974SRS150029\_contig\_number\_14676SRS892882\_contig\_number\_11299SRS893170\_contig\_number\_321SRS893172\_contig\_number\_11065SRS893187\_contig\_number\_20423SRS893252\_contig\_number\_9660SRS893253\_contig\_number\_443SRS893256\_contig\_number\_30SRS893259\_contig\_number\_3270SRS893270\_contig\_number\_21266SRS893279\_contig\_number\_17102SRS893285\_contig\_number\_163SRS893288\_contig\_number\_278SRS893292\_contig\_number\_7947SRS893295\_contig\_number\_528SRS893300\_contig\_number\_10356SRS893327\_contig\_number\_391SRS893341\_contig\_number\_5476SRS893342\_contig\_number\_21SRS893358\_contig\_number\_10649SRS893366\_contig\_number\_24196SRS893369\_contig\_number\_735SRS893373\_contig\_number\_20284SRS893378\_contig\_number\_619SRS893380\_contig\_number\_19SRS893382\_contig\_number\_7SRS893383\_contig\_number\_33193SRS971275\_contig\_number\_7636
328
821

1
2292941

SRS1041036\_contig\_number\_695
species


SRS140492\_contig\_number\_6365
162156
1
species


SRS1041143\_contig\_number\_31
1262739
1
species

species
291644
1

SRS074670\_contig\_number\_1769

1
371601

SRS046502\_contig\_number\_12421
species

species

SRS147377\_contig\_number\_4327
457390
1

species
2
818

SRS022713\_contig\_number\_1608SRS148784\_contig\_number\_4983


SRS020271\_contig\_number\_contig-100\_12186.12187
1
2292935
species

species

SRS058268\_contig\_number\_10
1
820

species
47678
2

SRS023583\_contig\_number\_contig-100\_1940.36266SRS077335\_contig\_number\_708


SRS015065\_contig\_number\_1672SRS105153\_contig\_number\_6323
46506
2
species


SRS077730\_contig\_number\_10106
469589
1
species

310298
1

SRS017375\_contig\_number\_82
species
